# No Evidence for Improved Associative Memory Performance Following Process-Based Associative Memory Training in Older Adults

**DOI:** 10.3389/fnagi.2016.00326

**Published:** 2017-01-09

**Authors:** Martin Bellander, Anne Eschen, Martin Lövdén, Mike Martin, Lars Bäckman, Yvonne Brehmer

**Affiliations:** ^1^Aging Research Center, Karolinska Institutet and Stockholm UniversityStockholm, Sweden; ^2^International Normal Aging and Plasticity Center (INAPIC), University of ZurichZurich, Switzerland; ^3^University Research Priority Program “Dynamics of Healthy Aging”, University of ZurichZurich, Switzerland; ^4^Department of Psychology, University of ZurichZurich, Switzerland; ^5^Otto Hahn Research Group on Associative Memory in Old Age, Max Planck Institute for Human DevelopmentBerlin, Germany

**Keywords:** associative memory, older adults, cognitive training, transfer, episodic memory

## Abstract

Studies attempting to improve episodic memory performance with strategy instructions and training have had limited success in older adults: their training gains are limited in comparison to those of younger adults and do not generalize to untrained tasks and contexts. This limited success has been partly attributed to age-related impairments in associative binding of information into coherent episodes. We therefore investigated potential training and transfer effects of process-based associative memory training (i.e., repeated practice). Thirty-nine older adults (*M*_age_ = 68.8) underwent 6 weeks of either adaptive associative memory training or item recognition training. Both groups improved performance in item memory, spatial memory (object-context binding) and reasoning. A disproportionate effect of associative memory training was only observed for item memory, whereas no training-related performance changes were observed for associative memory. Self-reported strategies showed no signs of spontaneous development of memory-enhancing associative memory strategies. Hence, the results do not support the hypothesis that process-based associative memory training leads to higher associative memory performance in older adults.

## Introduction

The decline of cognitive functioning in old age has a negative impact on individuals’ well-being and independence (Park et al., 2002). Specific attention has been devoted to the study of age-related changes in episodic memory (Tulving, 1972), the conscious remembrance of events situated in time and space. Longitudinal studies have shown that episodic memory functioning decreases in old age, starting on average around the age of 60 (Rönnlund et al., 2005; Schaie, 2005). Episodic memories are often complex and require both memory for single units of information (item memory) and memory for associations between these units (associative memory; Chalfonte and Johnson, 1996; Davachi, 2006). Generation of associations can take place at different levels (e.g., between features of an item, between different items, between items and context features) and include different types of information (e.g., verbal, visual or spatial information). Although younger adults in general outperform older adults in episodic memory, it has been shown with a range of materials that this difference is more pronounced in associative compared to item memory (e.g., Schacter et al., 1991; Naveh-Benjamin, 2000; Yonelinas, 2002; Old and Naveh-Benjamin, 2008). Performance differences between item and associative memory might result from the more elaborate encoding and retrieval processes that associative memory requires. The two-component framework of episodic memory across the lifespan (Shing et al., 2008, 2010, 2016) proposes that lower episodic memory performance in old age reflects senescent decline in two separate, but highly interactive components: a strategic and an associative component. The strategic component refers to cognitive control processes which aid and regulate memory functions (Simons and Spiers, 2003) and the associative component refers to binding mechanisms that integrate features of episodes into coherent representations (Treisman, 1996; Zimmer et al., 2006).

So far, episodic memory interventions in older adults have mainly targeted the strategic component of episodic memory by strategy-based training. Here, individuals are instructed and trained in a new memory strategy facilitating encoding and retrieval of information. Meta-analyses summarizing this research have shown that these interventions induce small to medium performance gains in the trained tasks (Verhaeghen et al., 1992; Gross et al., 2012). However, older adults seem to profit less than younger adults and children from such strategy-based interventions mostly because of deficits in the associative component of episodic memory (Stigsdotter Neely and Bäckman, 1993; Brehmer et al., 2007, 2008; Shing et al., 2010; see also Naveh-Benjamin et al., 2007). In Brehmer et al. (2007), children, younger adults and older adults learned and practiced an imagery-based mnemonic technique involving the creation and retrieval of associations between words and location cues. Older adults and children showed similar and lower baseline performance than younger adults in associative memory and profited to a similar degree from the instruction in the mnemonic technique, targeting the strategic component of episodic memory. However, children profited more than older adults from practice of this mnemonic technique, focusing on binding words and locations cues with images, probably because of older adults’ deficits in the associative component of episodic memory. In addition, strategy-based training seems to be very specific and to not yield transfer even to other untrained episodic memory tasks (for reviews, see Lustig et al., 2009; Eschen, 2012; Brehmer et al., 2014; but see Carretti et al., 2007; Bailey et al., 2014).

An alternative training approach is process-based training which aims to increase the efficiency of basic cognitive processes through extensive repetitive practice without providing explicit strategy instructions (Willis and Schaie, 2009; Lövdén et al., 2010; Brehmer et al., 2014). As of yet, most process-based interventions in older adults have practiced working memory or executive functions. A recent meta-analysis of this research (Karbach and Verhaeghen, 2014) points to more promising training and transfer effects of process-based than strategy-based training in older adults with medium to large performance gains in the trained tasks, small performance gains in untrained working memory or executive function tasks (near transfer) and untrained tasks measuring other abilities (far transfer), and similar training and transfer effects in younger and older adults. However, Melby-Lervåg and Hulme (2016) have criticized this meta-analysis for methodological reasons. They also re-analyzed studies on transfer effects of working memory training regimes to nonverbal reasoning included in Karbach and Verhaeghen (2014) finding small transfer effects only in studies with passive control groups, but no transfer in studies with active control groups.

With regard to associative memory, to our knowledge, only one process-based training regime in older adults has been investigated: the repetition-lag procedure (Jennings and Jacoby, 2003). In this training regime, word recognition is assessed with test lists in which distractor words are presented twice with performance-related increase in the number of intervening words between distractor repetitions, thus requiring retrieval of associations between words and the temporal context in which they appeared (study or test list). Although the repetition-lag procedure has led to large performance gains in the trained tasks, compared to passive (Jennings et al., 2005) and active control groups practising item recognition (Jennings et al., 2005; Stamenova et al., 2014), there was little evidence for transfer assessed by a range of untrained associative and item memory, working memory and speed tasks. The only clear transfer effect across studies was found to one untrained working memory task (Jennings et al., 2005) and performance gains in the trained tasks were not predicted by baseline episodic memory, but by baseline working memory performance (Stamenova et al., 2014). Hence, these studies indicate that the repetition-lag procedure practices working memory rather than episodic memory processes. Consequently, it is still unclear whether process-based training of the associative component of episodic memory in older adults improves associative binding processes and yields transfer to untrained cognitive abilities.

The main goal of the present study was to investigate potential training and transfer effects of several weeks of process-based associative memory training in a sample of older adults. The training was constructed without providing individuals with specific encoding and retrieval instructions (i.e., without tapping the strategic component of episodic memory). In line with previous process-based training regimes, we included a large amount of repeated practice trials using different and varying task materials. This should increase the need to bind two unrelated items, and hence tap the associative component or processes related to binding of items into a coherent episode directly. The scope of transfer was assessed with three associative memory, three visuospatial memory and three reasoning tasks administered before and after training. The three cognitive abilities were selected to represent near, intermediate and far transfer (see Noack et al., 2009 for further information regarding the classification of transfer tasks). The associative memory tasks measured item-item memory as practiced in the training tasks, but included different stimulus material. The visuospatial memory tasks also assessed associative memory, but required item-context binding instead of item-item binding. The reasoning tasks were selected because the ability to create stable associations between information units facilitates the construction and manipulation of new structural representations required for reasoning (Oberauer et al., 2007). Latent variable studies have shown that associative memory predicts variance in reasoning above and beyond working memory and speed in younger adults (Kaufman et al., 2009) and in samples covering most of the adult lifespan (Tamez et al., 2012). We used three instead of only one task per outcome ability and aggregated their scores to composite scores to avoid that observed transfer effects were only driven by task-specific surface commonalities between training and transfer tasks (e.g., material or response modality, see Lövdén et al., 2010; Shipstead et al., 2012).

Based on previous research on process-based training in older adults, we expected that individuals receiving associative memory training in comparison to individuals receiving item memory (i.e., active control group), would improve linearly in associative memory performance across the training period. In addition, we expected the process-based associative memory training to induce at least near transfer to untrained associative memory tasks and possibly also intermediate transfer to visuospatial memory and far transfer to reasoning.

## Materials and Methods

### Participants

The initial study sample consisted of 52 retired community dwellers living in Zurich (26 randomly assigned to the experimental group and 26 to the active control group, stratified by gender). They all fulfilled the inclusion criteria: (a) age between 65 and 75 years; (b) retirement from work; (c) native German speaker; and (d) having access to a computer with internet connection and basic knowledge in handling the computer. They did not have any of the following exclusion criteria: (a) previous or current neurological and psychiatric disorders or substance abuse negatively affecting brain function; (b) participation in another memory training study; (c) prior knowledge of memory strategies. Five participants had to be excluded as they failed cognitive screening tests (see description below; three of the experimental, two of the control group). In addition, two experimental group participants dropped out because of time conflicts. Of the remaining participants, two (one of each group) had to be excluded due to technical problems during training and four (one of the experimental, three of the control group) due to failures in training compliance (less than 30% correct answers in all distractor phase trials of the intervention, see description below). Thus, the final sample consisted of 39 individuals: 19 in the experimental group and 20 in the control group.

All participants gave written informed consent and were paid 40 CHF after completion of the screening session and 100 CHF after completion of the other study parts. Descriptive data of the final sample are summarized in Table 1.

**Table 1:** **Demographic variables, screening and descriptive measures for the two intervention groups**.

	Associative memory training group (*n* = 19) *M* (*SD*)	Active control group (*n* = 20) *M* (*SD*)	Effect size (Cohen’s *d*)
**Demographic variables**
Age	69.46 (2.65)	68.14 (2.47)	0.51
Years of education	14.71 (2.57)	14.80 (2.22)	−0.04
Gender (*n* male/*n* female)	12/7	10/10
**Screening measures**
MMSE	29.63 (0.60)	29.50 (0.61)	0.22
DSST	47.47 (8.66)	46.85 (7.40)	0.08
CES-D	5.21 (2.10)	4.55 (4.51)	0.19
**Descriptive measures**
Spot a word (crystallized intelligence)	27.37 (3.86)	27.81 (3.00)	−0.13
CVLT (correct immediate free recall total)	13.66 (1.32)	13.12 (1.47)	0.39
d2 (selective attention)	129.78 (21.71)	133.26 (24.17)	−0.15
Digit sorting	8.17 (2.79)	6.65 (2.16)	0.61
(working memory)

*Note. CVLT, California Verbal Learning Test; d2, d2 Test of Attention (Brickenkamp, 2002); DSS, Digit Symbol Substitution Test; MMSE, Mini Mental State Examination*.

### Study Design

The experimental (associative memory) and the control (item memory) interventions comprised 24 training sessions across 6 weeks (four sessions per week). Participants trained at home on their personal computers. To detect potential training and transfer effects of the interventions, a cognitive test battery was performed within 2 weeks before the start of the interventions (pre training) and in the week after intervention completion (post training).

Before the pre-training session, participants were invited to a screening session, in which a large cognitive battery was completed and participants filled out questionnaires regarding subjective health and lifestyle. In addition, individuals were screened for cognitive deficits or dementia with the Mini-Mental State Examination (MMSE; Folstein et al., 1975) and the Digit Symbol Substitution Test (DSST; Wechsler, 1981) as well as for clinically relevant depressive episodes with the German version of the Center for Epidemiological Studies Depression Scale (CES-D; Radloff, 1977). Participants were excluded from the study if they scored lower than 28 on the MMSE, lower than 35 on the DSST, and higher than 20 on the CES-D. The final study sample was invited to an individual 1-h introductory session in which they were familiarized with their training regime. The study was double blinded: neither the participants nor the experimenters conducting the screening, pre-training, and post-training sessions were aware of group assignments. Participants were recruited for a study comparing the effects of different types of memory training, but were not informed about the number or nature of the different training conditions.

### Training

The training was conducted at home using the open-source Java-based Tatool software (von Bastian et al., 2013). Individuals were instructed to train in a quiet room without interruptions (e.g., turning of cell phones). After each training session, data were automatically uploaded to a web server. Automatized online analyses permitted the detection of irregularities (e.g., accuracy below chance level), thus allowing for constant monitoring of participants’ training compliance. Participants were informed that the training software permitted the completion of only one session per day and that they would be contacted by e-mail or phone in case of deviations from the training schedule (i.e., four training sessions per week). To further enhance training commitment, they received weekly motivational e-mails.

The experimenters monitoring training compliance could also be contacted by participants in case of technical difficulties. To ensure that all participants were able to use the training software and to complete the training tasks, they practiced the installation of the software and completed a short version of the first training session. In addition, participants received a manual with step-by-step software installation instructions and detailed information about the training procedures.

#### Training Motivation and Affect

At the beginning of each training session, participants rated their current training motivation on a 5-point Likert scale (1 = “not at all motivated”, 5 = “very motivated”) and their current arousal and emotional valence on 9-point Likert scales using self-assessment manikins (Bradley and Lang, 1994; arousal:1 = “calm, relaxed”, 9 = “excited, stimulated”; valence: 1 = “annoyed, sad”, 9 = “happy, hopeful”).

#### Training Tasks

In each of the 24 sessions of the experimental and control training, participants practiced three memory tasks. The order of the three tasks within a session was randomly assigned, but such that they were equally distributed across the 24 sessions. The order was the same for all participants. Each task consisted of five trials. In the beginning of each training task, participants could complete an optional practice trial. Each task trial contained an encoding, a distractor and a retrieval phase.

During the *encoding phase* in both interventions, object-word pairs were presented consecutively for 6 s each in the middle of the screen (word below the object) with an ISI of 0.5 s. Instructions were to encode the pairs as well as possible. For the three tasks in each training session, object-word pairs from the following three category combinations were used: (a) jewelry and names; (b) dishes and geography; and (c) clothes and food. For each of the three category combinations, four different subcategory combinations were generated from which the stimulus material for the three training tasks in the first, second, third and fourth training session in each of the six training weeks was picked. For example, in the first training session of each week, participants worked on three training tasks presenting the following types of object-word pairs: (a) rings and contemporary female first names; (b) cups and countries; and (c) sweaters and meals (see Table 2 for the three subcategory combinations in each of the four training sessions per week). Thus, participants trained on 12 different subtasks each week. To establish some practical relevance, a specific cover story for each of the 12-subcategory combinations was provided (e.g., “You help a friend who owns a goldsmith workshop by taking new orders. To avoid confusion, please try to remember which young lady came along with which ring.”).

**Table 2:** **Stimulus materials for the 12 training tasks**.

Sessions	Task 1		Task 2		Task 3	
	Jewelry (Object)	Names (Word)	Dishes (Object)	Geography (Word)	Clothes (Object)	Food (Word)
1	Rings	Contemporary female names	Cups	Countries	Sweater	Meals
2	Watches	Contemporary male names	Plates	Animals	Pants	Eatable plants
3	Glasses	Old-fashioned male names	Vases	Plants	Shoes	Drinks
4	Earrings	Old-fashioned female names	Bowls	Cities	Bags	Sweets

The *distracter phase* in both interventions consisted of 30 s of simple arithmetic tasks to prevent that the encoded pairs were actively held in working memory. The arithmetic tasks involved subtraction or addition of three one- or two-digit numbers. Participants had to type in their results and were provided with feedback as to whether these were correct or not.

In the *retrieval phase* of the associative memory intervention, the encoded objects were presented in the middle of the screen and participants had to type in the corresponding word from the encoded object-word pairs (associative cued recall). In contrast, in the retrieval phase of the control intervention, participants were presented with single words or objects either from the encoding phase or new ones (half old and half new stimuli, half of the old and new stimuli were objects and the other half words). They had to indicate by pressing two separate keys whether or not they had seen the word/object before (item recognition). In both interventions, retrieval was self-paced with a maximum time of 20 s. Retrieval was followed by confidence ratings assessed using a three-point scale (“very sure”, “quite sure” and “not sure”) by pressing three corresponding keys. The outcome measure for both the associative and item memory training tasks was percentage of correct answers.

The associative memory intervention was adaptive. Based on performance during training, task difficulty was adjusted by increasing or decreasing the number of pairs that had to be remembered by one. If percentage of correctly recalled pairs in three consecutive trials of a training task was above 70%, task difficulty was increased and if performance was below 50%, task difficulty was decreased in the next trial of the task. All participants started the first session on the lowest level of difficulty with four stimulus pairs. In contrast, the active control group worked on a fixed and low level of difficulty (eight stimulus pairs) across all training sessions.

Object stimuli were photographs of real objects. For each stimulus subcategory, 190 different stimuli were available. These were drawn randomly with the restriction that different stimuli were used in the five trials of each training task in each session.

#### Feedback

Several types of feedback were included in the software. After each training task, the percentage of correct responses across the five trials in both interventions and the current level of task difficulty in the associative memory intervention were presented. Moreover, in the associative memory intervention, a smiley was displayed if percentage of correct responses was above 70% in three consecutive trials of a training task. At the end of each training session, with graphical illustrations, for each of the five trials of all three training tasks, accuracy in both interventions and additionally level of task difficulty in the associative memory intervention were presented.

A training session including login and feedback lasted on average 37.07 min (*SD* = 9.57) in the associative memory intervention and 33.39 min (*SD* = 2.31) in the control intervention.

### Criterion and Transfer Tasks

We used three tasks for the assessment of each of the four training and transfer abilities (item memory, associative memory, visuo-spatial memory and reasoning). We used three instead of only one task per ability and aggregated their scores into composites to avoid that potential transfer effects were driven by task-specific surface commonalities between training and transfer tasks (e.g., material or response modality, see Lövdén et al., 2010; Shipstead et al., 2012). The same tasks were used pre and post training. The order of the tasks was counterbalanced across the four transfer abilities and was the same for all participants at pre and post training. Associative memory and item memory were assessed within the same tasks.

#### Associative Memory and Item Memory (Criterion/Near Transfer)

The three associative and item memory tests were structured similarly, but contained different materials (modified according to Naveh-Benjamin, 2000). The associative memory tests tapped the process that was trained during associative training. Because memory for item information is required also for learning pair information, this ability could improve with training and can therefore be viewed as a measure of near transfer. During encoding, 44 pairs were presented consecutively for 6 s each and participants were instructed to encode the stimuli as pairs (the first and last two pairs were discarded from analysis to control for primacy and recency effects). After encoding, a distracter task was included in which participants had to count backwards from a 3-digit number in steps of 3 for 30 s. Afterwards, they had to type in the last number. This phase was followed by two self-paced recognition tasks: one associative and one item recognition task. In the associative recognition task, participants saw 20 pairs. Half of the pairs had been previously presented in the encoding phase (old), the other half was composed of two stimuli that had appeared in the encoding phase, but not together (rearranged). Participants had to indicate if they had seen a particular pair during encoding or not by pressing two corresponding keys within 5 s. In the item recognition task, participants saw 40 single items of which 20 had been studied during encoding and 20 were new. Ten new and 10 old items were from the first stimulus category of the pairs and 10 new and 10 old items were from the second stimulus category. Participants had to indicate if they had seen the stimuli in the encoding phase or not by pressing two keys within 5 s. Each of the 88 encoded stimuli appeared only in one of the two retrieval tests. After retrieval, confidence ratings were acquired using a three-point scale (“very sure”, “quite sure” and “not sure”) by pressing three corresponding keys within 5 s. The materials in the three tasks was: (a) German words—Malay words; (b) pictures of cups—pictures of watches; and (c) pictures of lamps—pictures of chairs. The number of hits minus number of false alarms (H−FA) served as measure of performance. In the beginning of each task, participants were provided with a practice trial with four pairs. Across the three memory tasks, eight different orders of the associative recognition and item recognition tests within and across the three tasks were possible. These were taken into account during randomization and counterbalanced across both intervention groups. For all participants, the order of the associative and item recognition tests within each memory task was the same pre and post training. Maximum scores were 40 in both the associative and item memory tasks.

#### Visuospatial Memory (Intermediate Transfer)

These tasks also assessed associative memory, but required item-context binding instead of item-item binding. The three visuospatial memory tests of the Berlin Intelligence Scale (BIS; Jäger et al., 1997) were used. In the first task (Orientation Memory), participants had 90 s to encode the locations of 27 marked buildings on a map. Directly afterwards, the map was presented without markings and participants had 90 s to mark the encoded buildings. In the second task (Remembering Paths), 30 s were given to encode a path from someone’s home to that person’s workplace on a map (30 path sections). Then participants were given an untagged map and had 40 s to reproduce the encoded path. In the third task (Company Logos), 20 company logos in differently shaped frames had to be encoded within 50 s. Afterwards, the logos were presented with four frames each and participants had to choose the correct frames within 90 s. The performance measure was the number of correctly retrieved buildings, path sections, or frames. Maximum numbers of points were 27, 30 and 20, respectively.

#### Reasoning (Far Transfer)

The reasoning tasks were selected, because the ability to create stable associations between information units facilitates the construction and manipulation of new structural representations required for reasoning (Oberauer et al., 2007). Three out of four subtasks measuring logical reasoning of the Kit of Factor-Referenced Cognitive Tests (Ekstrom et al., 1976) were used. In the first task (Deciphering Languages), an item included three German phrases along with translations in an artificial sign language. Afterwards, five new German phrases and their translations in this sign language were presented and participants had to choose the correct translation among the five alternatives. The task contained 12 items and the maximum time allowed was 8 min. In the second task (Diagramming Relationships), participants had to select the correct out of five diagrams that illustrated the relationships among three different sets of things. The task comprised 15 trials and had a time limit of 4 min. In the third task (Interference Test), participants were given one or two statements and had to choose the correct conclusion that could be deduced from five alternatives. Ten items had to be completed in 6 min. The performance measure in all three tasks was number of correct responses − number of errors/4. Maximum scores were 12, 15 and 10, respectively.

#### Memory Strategies

At the end of the pre- and post-training assessments, participants filled out a strategy questionnaire, in which they were asked to describe how they tried to remember the pairs in each of the three associative and item memory outcome tasks in an open-end format. These strategies were coded by two independent raters into three broad categories (see Dunlosky and Hertzog, 1998), namely associative (connecting two items of a pair semantically or visually), item (highlighting distinct features of the stimuli separately) and shallow (rote repetition, focusing on graphical aspects of the words, rehearsal). Participants could report using these strategies in any combination (e.g., using one of them exclusively or combining some of them). Each participant received a score for each of the categories, denoting the percentage of used strategies in the respective category relative to all strategies reported by the individual (see Brehmer et al., 2016; and Shing et al., 2016; for a similar approach).

### Statistical Analysis

The scores of all cognitive tasks were *z*-standardized. The motivation for the use of *z*-scores is foremost based on theoretical consideration and earlier research. Since the sample in this study is small, the correlations between tasks are unstable, even though we know from earlier research that the tasks tap into the same construct (e.g., Süß et al., 2002; Schmiedek et al., 2010). Hence, we used aggregated scores to attenuate some of the measurement error in the task scores and increase reliability of the measures. The correlations among the BIS tasks ranged between 0.0 and 0.4, and the KIT tasks between 0.1 and 0.5, the associative memory tasks between 0.0 and 0.2 and item memory tasks between 0.2 and 0.4), which is lower than expected, but possible in studies with these small sample sizes. Next, the *z-scores* of the three tasks assessing each of the four outcome abilities (i.e., item memory, associative memory, visuospatial memory and reasoning) were aggregated by calculating the means of the tasks. The training/transfer effects were analyzed with separate 2 (group: experimental, control) × 2 (time: pre training, post training) mixed analyses of variance (ANOVA) on the mean *z*-scores for each of the abilities. Strategy use was analyzed using 2 (group: experimental, control) × 2 (time: pre training, post training) mixed ANOVAs on the calculated associative, non-associative and shallow strategy scores separately. The threshold for significance was *p* < 0.05. Training motivation and affect was analyzed with a 2 (group: experimental, control) × 24 (training session) mixed ANOVA with motivation, arousal and valence ratings as separate dependent variables.

## Results

### Training Motivation and Affect

Table 3 displays the mean training motivation, arousal and valence ratings across the 24 training sessions in both intervention groups. Mean training motivation ratings were high, mean arousal ratings moderate and mean valence ratings positive in both groups. For all measures, the main effect of group was not significant (*F*s < 1). The main effect of time was significant for motivation (*F*_(1,23)_ = 2.53, *p* = 0.004, $\eta_{\text{p}}^{2}$ = 0.06) and valence (*F*_(1,23)_ = 2.49, *p* = 0.007, $\eta_{\text{p}}^{2}$ = 0.06), indicating that motivation and valence of participants decreased slightly across time. However, the two intervention groups did not differ in this change, as no group × training session interaction was observed (*F*s < 1).

**Table 3:** **Mean motivation, valence, and arousal ratings across the 24 training sessions in the two intervention groups**.

	Associative memory training group *M* (*SD*)	Active control group *M* (*SD*)
Motivation (scale: 1–5)	3.59 (0.76)	3.66 (0.80)
Valence (scale: 1–9)	6.70 (1.08)	6.90 (1.08)
Arousal (scale 1–9)	4.74 (1.52)	4.53 (1.38)

### Trained Associative Memory Tasks

**Figure 1:**
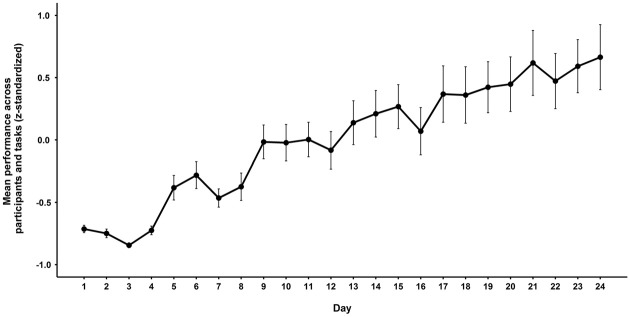
**Mean daily performance (*z*-standardized) averaged across trained tasks and participants of the associative memory-training group during the 6 weeks of training.** Error bars represent one standard error around the means.

As our training was adaptive for the associative memory group only, it is not possible to investigate changes on the trained tasks *per se* by comparing the two groups. However, training progress in the adaptive group can be evaluated (see Figure 1). On average, trained individuals increased their task difficulty level across the 6 weeks of training, which can be taken as an indicator that the individuals improved their performance during the training process.

### Item-Associative Memory (Criterion/Near Transfer)

**Figure 2:**
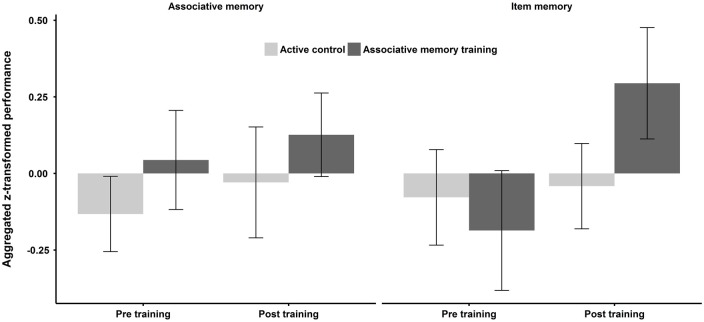
**Aggregated *z*-transformed performance across the three associative memory tasks and the three item memory tasks separately used as criterion tasks to assess training gains separately for the two intervention groups (i.e., associative memory training and active controls) before and after training.** Error bars represent one standard error around the means.

There were no significant effects of group, time, or their interaction on associative memory (all *F*s < 1). For item memory there was no main effect of group (*F* < 1), but a main effect of time (*F*_(1,37)_ = 6.10, *p* = 0.02, $\eta_{\text{p}}^{2}$ = 0.14), indicating that participants’ performance in the item memory tasks across both groups improved between pre and post training. Additionally, there was a group × time interaction (*F*_(1,37)_ = 4.49, *p* = 0.04, $\eta_{\text{p}}^{2}$ = 0.11), with the experimental group improving more than the control group from pre to post training. Figure 2 displays the associative memory and item memory composite scores in each group pre and post training.

### Visuospatial Memory (Intermediate Transfer)

There was no main effect of group for visuospatial memory (*F* < 1). There was a main effect of time (*F*_(1,37)_ = 9.21, *p* < 0.01, $\eta_{\text{p}}^{2}$ = 0.20), indicating that performance improved after training. There was no significant group × time interaction, *F*_(1,37)_ = 1.15, *p* = 0.29, suggesting that the training-related gains generalized across groups.

### Reasoning (Far Transfer)

There was no main effect of group (*F* < 1). There was a main effect of time (*F*_(1,37)_ = 8.74, *p* < 0.01, $\eta_{\text{p}}^{2}$ = 0.19), indicating that participants’ performance in the reasoning tasks across experimental groups improved from pre to post training. There was no group × time interaction (*F* < 1).

Table 4 provides the average composite scores for each of the four abilities in the two groups before and after training, and Table 5 provides the raw data for all transfer tasks.

**Table 4:** **Average *z*-transformed composite scores for each of the outcome abilities before and after training and the effect size of the change (*M*_post_ − *M*_pre_/*SD*_pooled_) in the two intervention groups**.

	Associative memory training group			Active control group		
	Pre training *M* (*SD*)	Post training *M* (*SD*)	Effect size	Pre training *M* (*SD*)	Post training *M* (*SD*)	Effect size
Item memory	−0.19 (0.85)	0.29 (0.79)	0.58	−0.08 (0.70)	−0.04 (0.62)	0.06
Associative memory	−0.04 (0.70)	0.13 (0.59)	0.13	−0.13 (0.55)	−0.03 (0.81)	0.15
Spatial memory	−0.23 (0.68)	0.18 (0.55)	0.67	−0.07 (0.65)	−0.12 (0.76)	0.28
Reasoning	−0.03 (0.73)	0.19 (0.87)	0.28	−0.20 (0.66)	−0.05 (0.64)	0.38

**Table 5:** **Average raw scores (Means (*M*) and Standard deviations (*SD*)) for each of the task for the four outcome abilities before and after training**.

		Associative memory training group		Active control group	
Domain	Task	Pre training *M (SD)*	Post training *M (SD)*	Pre training *M (SD)*	Post training *M (SD)*
Associative memory	CW	0.06 (0.12)	0.05 (0.13)	0.03 (0.12)	0.1 (0.14)
	GM	0.19 (0.14)	0.21 (0.16)	0.14 (0.12)	0.16 (0.11)
	LC	0.06 (0.1)	0.08 (0.11)	0.07 (0.11)	0.02 (0.13)
Item memory	CW	0.13 (0.07)	0.18 (0.09)	0.16 (0.1)	0.14 (0.08)
	GM	0.22 (0.11)	0.26 (0.08)	0.24 (0.06)	0.26 (0.08)
	LC	0.27 (0.09)	0.29 (0.09)	0.24 (0.09)	0.25 (0.07)
Reasoning	Deciphering language	4.51 (1.51)	4.87 (1.81)	4.64 (1.58)	4.53 (1.78)
	Interference test	3.34 (2.22)	3.51 (1.99)	2.39 (2.19)	3.34 (1.89)
	Diagraming relationships	5.43 (2.73)	6.53 (3.46)	5.09 (2.97)	6.14 (2.46)
Spatial memory	Orientation memory	10.32 (4.37)	13.11 (2.42)	11.7 (3.21)	13.35 (2.74)
	Company logos	6.21 (2.25)	7.21 (2.25)	6.5 (2.61)	7.1 (3.24)
	Remembering path	12.89 (4.95)	13.05 (4.55)	12.75 (3.42)	12.2 (5.24)

*Note. CW, Cup-Watches; GM, German-Malay; LC, Lamps-Chairs Item-Associative Memory Tasks. The reasoning tasks are taken from the KIT of Factor-Referenced Cognitive Tests, the spatial memory tests are taken from the Berlin Intelligence Scale (BIS)*.

### Subjective Report of Strategy Use

**Figure 3:**
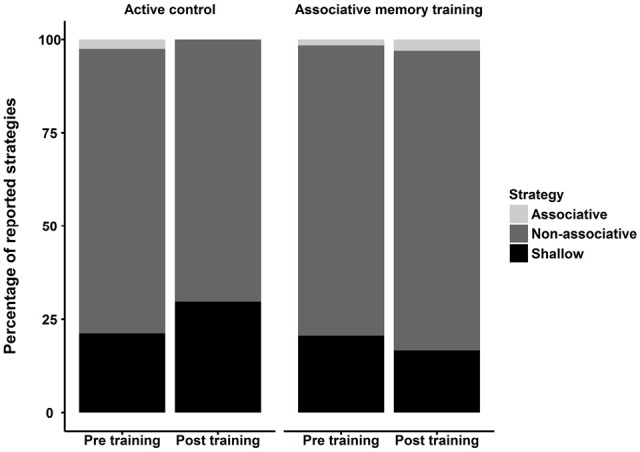
**Percentage of reported strategies applied in the item-associative memory tasks separately for the two intervention groups (i.e., associative memory training and active controls) before and after training.** Reported strategies were grouped into associative, non-associative and shallow strategies.

To investigate whether the two groups differentially developed associative memory strategies during training, we asked participants to report which memory strategies they had applied in the three item-associative memory tasks pre and post training. Reported strategies were sorted into associative, non-associative and shallow memory strategies. In general, participants were not applying associative strategies during task performance (associative memory training group: 1.3% of all reported strategies pre training, 1.9% post training; active control group: 2.3% pre training, 0% post training), but mostly used non-associative strategies (associative memory training group: 75.9% pre training, 78.8% post training; active control group: 78.3% pre training, 68.7% post training) or shallow strategies (associative memory training group: 22.8% pre training, 19.3% post training; active control group: 19.5% pre training, 31.3% post training; see Figure 3). There was no evidence that the two groups differed in strategy use; they did not differ at pre training in the type of strategies applied (*F* < 1) and no group × time interaction was observed (*p*s > 0.20). We rerun all analyses of transfer effects after excluding the three participants who used associative strategies at pretest, however, this did not alter the results substantially and all interpretations remained the same.

## Discussion

The goal of this study was to explore potential effects of process-based associative memory training on associative, item and spatial memory, as well as on reasoning. A group of older adults underwent 6 weeks of process-based adaptive associative memory training and their performance was compared to an active control group performing non-adaptive item-memory training for the same amount of time. Individuals in both groups improved performance in item memory, visuospatial memory (object-context binding) and reasoning as a function of training. A selective effect of associative memory training was only observed for item memory, where this group gained more than the control group from training. However, neither training-general (main effect of time) nor training-specific (time × group interaction) effects were observed for associative memory. These findings are in line with the only other process-based traning regime targeting associative memory processes in older adults, which also did not induce clear transfer to other untrained associative memory tasks nor to item memory, executive functioning, or speed tasks (Jennings et al., 2005; Stamenova et al., 2014). In line with previous studies we also found that older adults do not spontaneously generate associative strategies, which are often complex in nature (Dunlosky and Hertzog, 1998; Hertzog and Dunlosky, 2004; Dunlosky et al., 2005). Hence, our results do not support the notion that process-based associative memory training leads to higher associative memory performance in older adults when no encoding or retrieval instructions are provided.

There is behavioral and neural evidence that associative memory functioning under intentional encoding instructions depends on strategic processes (Naveh-Benjamin et al., 2009; Becker et al., 2015) but also on associative binding mechanisms (Moscovitch, 1992; O’Reilly and Norman, 2002; Simons and Spiers, 2003). The current data suggest that these binding mechanisms are difficult to train in a bottom-up fashion in aging. This apparent lack of modifiability of associative memory underscores the difficulty elderly persons have in binding pieces of information together.

Interestingly, training-specific effects were observed for item memory. Although the experimental group received associative memory training, the only measure on which they statistically differed from the controls across training was item memory. This is a challenging finding, as the control group trained on item memory. However, because associative memory also requires memory for the different items, it might be that the more demanding associative training regime improved item memory more by taxing processes that this form of memory relies on to a greater extent. In addition, time effects were observed for spatial memory, object-context binding, as well as for reasoning following both types of training; thus, parts of these improvements might be more than just test-retest effects, as the item-memory training group had the same encoding context as the associative training group; the only difference being the retrieval context, namely to recall associations or to make old/new judgments on item information. As all item and associative memory transfer tasks used recognition, this might have favored the control group. However, this is unlikely given that recall tests are more challenging than recognition tests. Because the encoding phase was the same for the training and the active control groups, the controls might still have incidentally encoded the material in an associative manner, thereby training that process, and leading to similar transfer for both groups. Of course, part of the expected training effects could come from the retrieval process. When forcing the memory system to retrieve information it might learn more effectively (Karpicke and Roediger, 2008) and this could potentially induce plastic changes more effectively than just recognition. On the other hand, even though the controls did item recognition, they might still recall the other item of the pair when presented with one item, thereby still training the associative component even though this is not required by the task. A no-contact control group could have been helpful to investigate if there are any specific transfer effects following this kind of process-based item and associative memory training.

A limitation of the study is that the gains in the trained tasks could not be assessed with the present design. This would have required participants of both groups doing the same tasks non-adaptively both before and after training. This would have helped to evaluate if there were any training gains from processes-based associative memory training on the actually trained tasks, which might be a prerequisite for transfer effects. However, visual inspection of the increase in task difficulty (an indirect indicator of performance improvements across training) showed that individuals generally increased the list length between two and three items depending on the task trained (see Figure 1). This indicates that, even though a direct examination of training-related gains is not possible in this study design, the data suggest that training had an effect, as participants were able to increase the task difficulty up to on average 60% correct responses. However, this line of reasoning has limitations. This is so because if subjects start at a difficulty level that is lower than what they actually could achieve then the training gains observed might just reflect adaptation to the individuals’ ability without actually inducing any improvement**.** Methodologically, it is important to note the small sample size in the two groups, which results in low power to detect group × time interactions. As there were large inter-individual differences in how much participants gained from training, the investigation of these inter-individual differences (e.g., training gains, training curves) need to be taken into account in future studies. In addition, future studies should consider alternative ways of modulating the associative load across the training process. Although we manipulated list length to stress the difficulty of associative memory, it might have been more effective to adjust the encoding time or to increase the associative load by increasing the number of to-be-associated items.

In addition, in this study we investigated only the training gains and potential transfer effects of process-based associative memory training in comparison to an active control group receiving item-memory training. We did not investigate the durability of these effects over time. Even though, we were not able to detect the expected transfer effects, it would still have been interesting to investigate potential sleeper effects, namely training and transfer effects that are not observed directly after training however reveal only after some time.

To conclude, we did not find evidence for improvement in older adults’ associative memory performance following process-based training of the associative component of episodic memory. Future studies have to clarify whether associative binding processes can generally not be improved by repeated practice in older adults or if other types of process-based training are more successful.

## Ethics Statement

Data collection took place at Zurich University in 2006. No ethical permission was needed at that time if only behavioral data of healthy participants were assessed. Participants were informed about the study goals and the procedure. They were informed that they participation is voluntary and that they can terminate study participation without any negative consequences. Data were handled pseudo-anonymously. Participants were insured while performing tests at the University and received compensation for participation.

## Author Contributions

All authors are accountable for all aspects of the work in ensuring that questions related to the accuracy or integrity of any part of the work are appropriately investigated and resolved. All authors provided final approval of the version to be published. In addition, YB and AE provided substantial contributions to the conception and design of the work and were responsible for the acquisition, analysis and interpretation of the data. They critically revised and drafted partly the manuscript. MB was involved in data acquisition and analysis and drafted the first version of the manuscript. ML, MM and LB substantially contributed to the interpretation of the data and provided important intellectual feedback to the manuscript.

## Conflict of Interest Statement

The authors declare that the research was conducted in the absence of any commercial or financial relationships that could be construed as a potential conflict of interest.

## References

[B1] Bailey H. R. Dunlosky J. Hertzog C. (2014). Does strategy training reduce age-related deficits in working memory? Gerontology 60, 346–356. 10.1159/000356699 24577079 PMC4074452

[B56] von Bastian C. C. Locher A. Ruflin M. (2013). Tatool: a java-based open-source programming framework for psychological studies. Behav. Res. Methods 45, 108–115. 10.3758/s13428-012-0224-y 22723043

[B2] Becker N. Laukka E. J. Kalpouzos G. Naveh-Benjamin M. Bäckman L. Brehmer Y. (2015). Structural brain correlates of associative memory in older adults. Neuroimage 118, 146–153. 10.1016/j.neuroimage.2015.06.002 26054875

[B3] Bradley M. M. Lang P. J. (1994). Measuring emotion: the self-assessment manikin and the semantic differential. J. Behav. Ther. Exp. Psychiatry 25, 49–59. 10.1016/0005-7916(94)90063-9 7962581

[B4] Brehmer Y. Kalpouzos G. Wenger E. Lövdén M. (2014). Plasticity of brain and cognition in older adults. Psychol. Res. 78, 790–802. 10.1007/s00426-014-0587-z 25261907

[B5] Brehmer Y. Li S.-C. Müller V. von Oertzen T. Lindenberger U. (2007). Memory plasticity across the life span: uncovering children’s latent potential. Dev. Psychol. 43, 465–478. 10.1037/0012-1649.43.2.465 17352553

[B6] Brehmer Y. Li S.-C. Straube B. Stoll G. von Oertzen T. Müller V.. (2008). Comparing memory skill maintenance across the life span: preservation in adults, increase in children. Psychol. Aging 23, 227–238. 10.1037/0882-7974.23.2.227 18572999

[B7] Brehmer Y. Shing Y. L. Heekeren H. R. Lindenberger U. Bäckman L. (2016). Training-induced changes in subsequent memory effects: no major differences among children, younger adults and older adults. Neuroimage 131, 214–225. 10.1016/j.neuroimage.2015.11.074 26673112

[B8] Brickenkamp R. (2002). Test d2: Aufmerksamkeits-Belastungs-Test [Manual] (Vol. 9). Göttingen: Hogrefe.

[B9] Carretti B. Borella E. De Beni R. (2007). Does strategic memory training improve the working memory performance of younger and older adults? Exp. Psychol. 54, 311–320. 10.1027/1618-3169.54.4.311 17953152

[B10] Chalfonte B. L. Johnson M. K. (1996). Feature memory and binding in young and older adults. Mem. Cogn. 24, 403–416. 10.3758/bf03200930 8757490

[B11] Davachi L. (2006). Item, context and relational episodic encoding in humans. Curr. Opin. Neurobiol. 16, 693–700. 10.1016/j.conb.2006.10.012 17097284

[B12] Dunlosky J. Hertzog C. (1998). Aging and deficits in associative memory: what is the role of strategy production? Psychol. Aging 13, 597–607. 10.1037/0882-7974.13.4.597 9883460

[B13] Dunlosky J. Hertzog C. Powell-Moman A. (2005). The contribution of mediator-based deficiencies to age differences in associative learning. Dev. Psychol. 41, 389–400. 10.1037/0012-1649.41.2.389 15769194

[B14] Ekstrom R. B. French J. W. Harman H. H. Dermen D. (1976). Manual for Kit of Factor Referenced Cognitive Tests. Princeton, NJ: Educational Testing Service.

[B15] Eschen A. (2012). The contributions of cognitive trainings to the stability of cognitive, everyday, and brain functioning across adulthood. GeroPsych 25, 223–234. 10.1024/1662-9647/a000073

[B16] Folstein M. F. Folstein S. E. McHugh P. R. (1975). “Mini-mental state”. A practical method for grading the cognitive state of patients for the clinician. J. Psychiatr. Res. 12, 189–198. 10.1016/0022-3956(75)90026-6 1202204

[B17] Gross A. L. Parisi J. M. Spira A. P. Kueider A. M. Ko J. Y. Saczynski J. S.. (2012). Memory training interventions for older adults: a meta-analysis. Aging Mental Health 16, 722–734. 10.1080/13607863.2012.667783 22423647 PMC3430800

[B18] Hertzog C. Dunlosky J. (2004). Aging, metacognition, and cognitive control. Psychol. Learn. Motiv. 45, 215–251. 10.1016/S0079-7421(03)45006-8

[B19] Jennings J. M. Jacoby L. L. (2003). Improving memory in older adults: training recollection. Neuropsychol. Rehabil. 13, 417–440. 10.1080/09602010244000390

[B20] Jennings J. M. Webster L. M. Kleykamp B. A. Dagenbach D. (2005). Recollection training and transfer effects in older adults: successful use of a repetition-lag procedure. Neuropsychol. Dev. Cogn. B Aging Neuropsychol. Cogn. 12, 278–298. 10.1080/138255890968312 24428336

[B21] Jäger A. O. Süß H.-M. Beauducel A. (1997). Berliner Intelligenzstruktur-Test, BIS-Test. Form 4. Handanweisung. Göttingen: Hogrefe.

[B22] Karbach J. Verhaeghen P. (2014). Making working memory work: a meta-analysis of executive-control and working memory training in older adults. Psychol. Sci. 25, 2027–2037. 10.1177/0956797614548725 25298292 PMC4381540

[B23] Karpicke J. D. Roediger H. L. III (2008). The critical importance of retrieval for learning. Science 319, 966–968. 10.1126/science.1152408 18276894

[B101] Kaufman S. B. DeYoung C. G. Gray J. R. Brown J. Mackintosh N. (2009). Associative learning predicts intelligence above and beyond working memory and processing speed Intelligence 37, 374–382. 10.1016/j.intell.2009.03.004

[B24] Lustig C. Shah P. Seidler R. Reuter-Lorenz P. A. (2009). Aging, training and the brain: a review and future directions. Neuropsychol. Rev. 19, 504–522. 10.1007/s11065-009-9119-9 19876740 PMC3005345

[B25] Lövdén M. Bäckman L. Lindenberger U. Schaefer S. Schmiedek F. (2010). A theoretical framework for the study of adult cognitive plasticity. Psychol. Bull. 136, 659–676. 10.1037/a0020080 20565172

[B27] Melby-Lervåg M. Hulme C. (2016). There is no convincing evidence that working memory training is effective: a reply to Au et al. (2014) and Karbach and Verhaeghen (2014). Psychon. Bull. Rev. 23, 324–330. 10.3758/s13423-015-0862-z 26082279

[B28] Moscovitch M. (1992). Memory and working-with-memory: a component process model based on modules and central systems. J. Cogn. Neurosci. 4, 257–267. 10.1162/jocn.1992.4.3.257 23964882

[B29] Naveh-Benjamin M. (2000). Adult age differences in memory performance: tests of an associative deficit hypothesis. J. Exp. Psychol. Learn. Mem. Cogn. 26, 1170–1187. 10.1037/0278-7393.26.5.1170 11009251

[B30] Naveh-Benjamin M. Brav T. K. Levy O. (2007). The associative memory deficit of older adults: the role of strategy utilization. Psychol. Aging 22, 202–208. 10.1037/0882-7974.22.1.202 17385995

[B31] Naveh-Benjamin M. Shing Y. L. Kilb A. Werkle-Bergner M. Lindenberger U. Li S. C. (2009). Adult age differences in memory for name-face associations: the effects of intentional and incidental learning. Memory 17, 220–232. 10.1080/09658210802222183 18654927

[B32] Noack H. Lövdén M. Schmiedek F. Lindenberger U. (2009). Cognitive plasticity in adulthood and old age: gauging the generality of cognitive intervention effects. Restor. Neurol. Neurosci. 27, 435–453. 10.3233/RNN-2009-0496 19847069

[B33] O’Reilly R. C. Norman K. A. (2002). Hippocampal and neocortical contributions to memory: advances in the complementary learning systems framework. Trends Cogn. Sci. 6, 505–510. 10.1016/s1364-6613(02)02005-3 12475710

[B34] Oberauer K. Süss H.-M. Wilhelm O. Sander R. (2007). “Individual differences in working memory capacity and reasoning ability,” in Variation in Working Memory, eds Conway A. Jarrold C. Kane M. Miyake A. Towse J. (Oxford: Oxford University Press), 49–75.

[B35] Old S. R. Naveh-Benjamin M. (2008). Differential effects of age on item and associative measures of memory: a meta-analysis. Psychol. Aging 23, 104–118. 10.1037/0882-7974.23.1.104 18361660

[B36] Park D. C. Lautenschlager G. Hedden T. Davidson N. S. Smith A. D. Smith P. K. (2002). Models of visuospatial and verbal memory across the adult life span. Psychol. Aging 17, 299–320. 10.1037/0882-7974.17.2.299 12061414

[B37] Radloff L. S. (1977). The CES-D scale: a self-report depression scale for research in the general population. Appl. Psychol. Meas. 1, 385–401. 10.1177/014662167700100306

[B38] Rönnlund M. Nyberg L. Bäckman L. Nilsson L.-G. (2005). Stability, growth and decline in adult life span development of declarative memory: cross-sectional and longitudinal data from a population-based study. Psychol. Aging 20, 3–18. 10.1037/0882-7974.20.1.3 15769210

[B39] Schacter D. L. Kaszniak A. W. Kihlstrom J. F. Valdiserri M. (1991). The relation between source memory and aging. Psychol. Aging 6, 559–568. 10.1037/0882-7974.6.4.559 1777144

[B40] Schaie K. W. (2005). What can we learn from longitudinal studies of adult development? Res. Hum. Dev. 2, 133–158. 10.1207/s15427617rhd0203_4 16467912 PMC1350981

[B41] Schmiedek F. Lövdén M. Lindenberger U. (2010). Hundred days of cognitive training enhance broad cognitive abilities in adulthood: findings from the COGITO study. Front. Aging Neurosci. 2:27. 10.3389/fnagi.2010.00027 20725526 PMC2914582

[B42] Shing Y. L. Brehmer Y. Heekeren H. R. Bäckman L. Lindenberger U. (2016). Neural activation patterns of successful episodic encoding: reorganization during childhood, maintenance in old age. Dev. Cogn. Neurosci. 20, 59–69. 10.1016/j.dcn.2016.06.003 27434313 PMC6987717

[B43] Shing Y. L. Werkle-Bergner M. Brehmer Y. Müller V. Li S. C. Lindenberger U. (2010). Episodic memory across the lifespan: the contributions of associative and strategic components. Neurosci. Biobehav. Rev. 34, 1080–1091. 10.1016/j.neubiorev.2009.11.002 19896974

[B44] Shing Y. L. Werkle-Bergner M. Li S.-C. Lindenberger U. (2008). Associative and strategic components of episodic memory: a life-span dissociation. J. Exp. Psychol. Gen. 137, 495–513. 10.1037/0096-3445.137.3.495 18729712

[B45] Shipstead Z. Redick T. S. Engle R. W. (2012). Is working memory training effective? Psychol. Bull. 138, 628–654. 10.1037/a0027473 22409508

[B46] Simons J. S. Spiers H. J. (2003). Prefrontal and medial temporal lobe interactions in long-term memory. Nat. Rev. Neurosci. 4, 637–648. 10.1038/nrn1178 12894239

[B47] Stamenova V. Jennings J. M. Cook S. P. Walker L. A. S. Smith A. M. Davidson P. S. R. (2014). Training recollection in healthy older adults: clear improvements on the training task, but little evidence of transfer. Front. Hum. Neurosci. 8:898. 10.3389/fnhum.2014.00898 25477801 PMC4235376

[B48] Stigsdotter Neely A. Bäckman L. (1993). Maintenance of gains following multifactorial and unifactorial memory training in late adulthood. Educ. Gerontol. 19, 105–117. 10.1080/0360127930190202

[B49] Süß H. M. Oberauer K. Wittmann W. W. Wilhelm O. Schulze R. (2002). Working-memory capacity explains reasoning ability—and a little bit more. Intelligence 30, 261–288. 10.1016/s0160-2896(01)00100-3

[B50] Tamez E. Myerson J. Hale S. (2012). Contributions of associative learning to age and individual differences in fluid intelligence. Intelligence 40, 518–529. 10.1016/j.intell.2012.04.004

[B51] Treisman A. (1996). The binding problem. Curr. Opin. Neurobiol. 6, 171–178. 10.1016/S0959-4388(96)80070-5 8725958

[B52] Tulving E. (1972). “Episodic and semantic memory,” in Organization of Memory, eds Tulving E. Donaldson W. (New York, NY: Academic Press), 381–403.

[B54] Verhaeghen P. Marcoen A. Goossens L. (1992). Improving memory performance in the aged through mnemonic training: a meta-analytic study. Psychol. Aging 7, 242–251. 10.1037/0882-7974.7.2.242 1535198

[B55] Willis S. L. Schaie K. W. (2009). Cognitive training and plasticity: theoretical perspective and methodological consequences. Restor. Neurol. Neurosci. 27, 375–389. 10.3233/RNN-2009-0527 19847065 PMC3607292

[B53] Wechsler D. (1981). Manual for the Wechsler Adult Intelligence Scale, Revised. New York, NY: The Psychological Corporation.

[B57] Yonelinas A. P. (2002). The nature of recollection and familiarity: a review of 30 years of research. J. Mem. Lang. 46, 441–517. 10.1006/jmla.2002.2864

[B58] Zimmer H. Mecklinger A. Lindenberger U. (2006). Handbook of Binding and Memory Perspectives from Cognitive Neuroscience. Oxford: Oxford University Press.

